# Validation of the Formulas for Mechanical Power in Children and Proposal of the Concept of “Effective Mechanical Power”

**DOI:** 10.3390/jcm15051781

**Published:** 2026-02-26

**Authors:** María Ferrón-Vivó, Alicia Baza-Del-Amo, María J. Rupérez, Antonio Martínez-Millana, Ana M. Pedrosa, Roberto Tornero-Costa, Yolanda Rubio-Atienza, Marta Aguar-Carrascosa, Cristina Camilo, Alberto Medina, Vicent Modesto-i-Alapont

**Affiliations:** 1 Instituto de Ingeniería Mecánica y Biomecánica (I2MB), Universitat Politècnica de València, 46022 Valencia, Spain; mjrupere@upvnet.upv.es (M.J.R.); anpedsan@dimm.upv.es (A.M.P.); 2 Pediatric Intensive Care Unit, Anesthesiology Department, Hospital Universitari i Politècnic La Fe, 46026 Valencia, Spain; baza_ali@gva.es (A.B.-D.-A.); yolanda.rubio.atienza@gmail.com (Y.R.-A.); aguar_mar@gva.es (M.A.-C.); vicent.modesto@gmail.com (V.M.-i.-A.); 3 Instituto Universitario de Investigación de Aplicaciones de las Tecnologías de la Información y de las Comunicaciones Avanzadas (ITACA), Universitat Politècnica de València, 46022 Valencia, Spain; anmarmil@upv.edu.es (A.M.-M.); rotorcos@itaca.upv.es (R.T.-C.); 4 Pediatric Intensive Care Unit, Hospital de Santa María MHM, Unidade Locale de Saúde, 1649-035 Lisboa, Portugal; cristinacamilo.cc@gmail.com; 5 Pediatric Intensive Care Unit, Hospital Universitario Central Asturias, 33011 Oviedo, Spain; amedinavillanueva@gmail.com

**Keywords:** mechanical ventilation, pediatric intensive care, ventilator-induced lung injury, respiratory mechanics, respiratory distress syndrome, critical care

## Abstract

**Background/Objectives**: Recently, studies have emerged raising ergotrauma as a new explanation for ventilator-induced lung injury development, with the concept of inspiratory Mechanical Power (MP) as a single physical variable to estimate the contribution of different ventilatory parameters to lung damage. A high value of inspiratory MP is associated with risk of harming the respiratory system. Furthermore, we propose the concept of effective MP as the energy per minute that dissipates in the lungs, subtracting expiratory MP from inspiratory MP. Our objective is to validate the equations proposed for adults to estimate inspiratory MP in children, and to develop and validate equations to estimate effective MP. **Methods**: Prospective convenience sampling of 18 children undergoing mechanical ventilation in volume-controlled ventilation mode, admitted to the pediatric intensive care unit of a tertiary university hospital. Data of ventilation parameters, loops and curves were obtained electronically with the automated procedure provided by the device software and compared with the theoretical results of the equations. **Results**: Among the available equations for calculating inspiratory MP in adults under volume-controlled ventilation, the simplified Gattinoni and generalized Giosa equations provided the best estimates in children (R^2^ > 0.99). For expiratory MP, the extended equation proposed in this study showed the best agreement with experimental results (R^2^ = 0.9954). Finally, for effective MP, the simplified equation was the most accurate (R^2^ = 0.9950). **Conclusions**: This study validated existing inspiratory MP equations in pediatric patients and introduced the concept of effective MP, together with a bedside equation for its estimation. Future studies should determine effective MP thresholds associated with ventilator-induced injury.

## 1. Introduction

Approximately 5–10% of mechanically ventilated patients develop ventilator-associated events [1]. Ventilator-induced lung injury (VILI) occurs when lungs are repeatedly subjected to excessive pressures and deformations, resulting in damage to the respiratory structures [2]. VILI was traditionally attributed to barotrauma, volutrauma, atelectrauma and biotrauma [3].

Since the 2000s, the concept of ergotrauma has been proposed to explain VILI from a physical perspective [4,5,6]. This approach integrates ventilatory settings and material science principles such as stress, strain, and energy transfer. In this context, Gattinoni et al. proposed the concept of Mechanical Power (MP) as a single physical quantity to estimate the contribution of different ventilatory parameters to lung damage. MP was defined as “the power delivered to the respiratory system, that is the amount of energy delivered to the respiratory system by the ventilator in the time unit (J/min)” [7]. A mathematical equation for estimating MP in inspiration (*MP_insp_*) was proposed for patients ventilated in volume-controlled (VC) mode [8]. This equation is based on bedside ventilatory parameters and a geometrical approach derived from the shape of the dynamic pressure–volume loop.

A high value of *MP_insp_* is associated with increased stress and strain in the lungs. Therefore, it has been associated with VILI and mortality in several multivariate analyses of mechanically ventilated patients with acute respiratory distress syndrome (ARDS) [9,10,11,12,13,14]. This association has also been reported in pediatric patients [15,16]. Therefore, efforts have focused on identifying *MP_insp_* thresholds associated with VILI, leading to the proposal of several *MP_insp_* equations. These include a simplified version of Gattinoni’s equation by Giosa et al. [17] and formulas for pressure-controlled (PC) ventilation proposed by Becher [18] and others [19,20], none of which have been validated in children.

**Figure 1:**
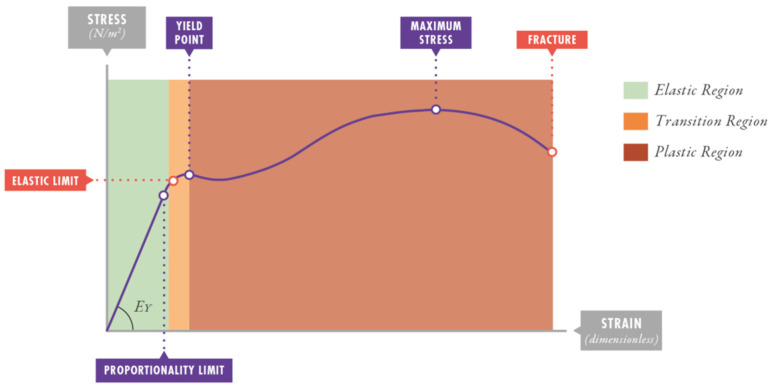
Graphic representation of the concept of resilience. Reprinted with permission from Copyright 2022, Villanueva A. M. et al. [25].

In materials science, resilience [21] is defined as the maximal capacity of any material to absorb energy when it is deformed elastically and to deliver this energy upon unloading. It is computed as the geometric area of the triangle below the initial linear elastic region of the stress–strain curve, up to the yield point. A graphic representation can be seen in Figure 1. In elastic bodies, deformations that remain within the limits of resilience do not result in permanent damage. Accordingly, as some authors have noticed [22,23,24], only the energy that exceeds this resilience threshold is what produces harm in the respiratory system. From a physical point of view, part of this *MP_insp_* is delivered as the expiratory MP (*MP_exp_*), the energy per minute recovered by the ventilator from the lungs in expiration. Therefore, measuring “Effective MP” (*MP_eff_*), defined as the difference between *MP_insp_* and *MP_exp_*, seems crucial, as it represents the energy per minute dissipated in the lungs. Further studies are needed to determine whether higher *MP_eff_* is clinically associated with lung injury or mortality and whether it provides a better explanation for VILI development.

The first objective of this study was to validate, in children ventilated in volume-controlled mode, previously published adult equations for *MP_insp_*. The second objective was to develop and validate a pediatric equation for estimating *MP_eff_*.

## 2. Materials and Methods

### 2.1. Study Population

During 5 random days from January to June 2023, a convenience sample of children admitted to the Pediatric Intensive Care Unit of a tertiary university hospital was selected. All children were ventilated in VC mode, no matter what the pathologies were, with a Getinge Servo U Ventilator (Getinge, Gothenburg, Sweden), and they were deeply sedated and receiving neuromuscular blockers if needed. Children were excluded if they suffered from a disease that tended to produce alteration of thoracic compliance, like open sternum, or if data were impossible to obtain electronically with the automated procedure provided by the device software. Parents of children admitted to our unit signed general informed consent for the use of their clinical data for research purposes. According to our institution ethics committee, further specific consent was not required for this study.

### 2.2. Experimental Measurements

**Figure 2:**
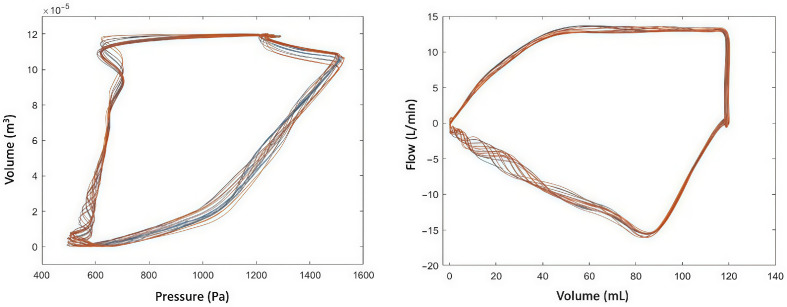
Example of the volume–pressure and flow–pressure loops obtained from the ventilator of a single patient in VC-CMV mode.

In every analysis, experimentally measured MP (either inspiratory, expiratory or effective) was used as the “gold standard” in the comparisons. Data were obtained from the device using the automated procedure that recorded instantaneous information from the pressure, volume, flow and time variables provided by the ventilator. Several respiratory cycles for 30 s intervals of recordings were obtained for each patient to average. Inspiratory and expiratory holding maneuvers were also applied to obtain static measurements of compliance, resistance, plateau pressure (*P_plateau_*) and total positive end-expiratory pressure (*PEEP*). Figure 2 shows an example of the dynamic pressure–volume (P-V) and flow–volume loops obtained from a single patient.

**Figure 3:**
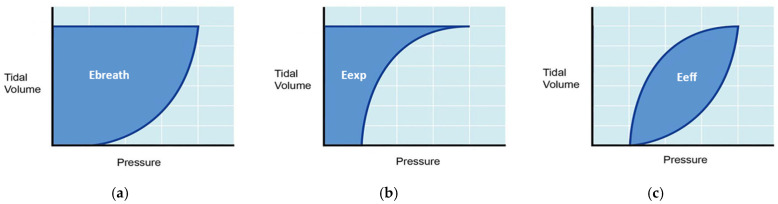
Graphical representation of (**a**) energy per breath in inspiration (E_breath_), (**b**) energy per breath in expiration (E_exp_) and (**c**) effective energy per breath (E_eff_).

All patient measurements were obtained from the ventilator as digital data files. The different P-V loops were plotted, and the areas under the curves were calculated using an executable program written in MATLAB^®^ R2022a (The MathWorks, Natick, MA, USA). Experimental measures of the three types of energy per breath (inspiratory, expiratory and effective) were obtained breath by breath from the downloaded raw data. Numerical integration was performed using the trapezoidal method implemented through the MATLAB function *trapz*. This corresponded to the area between the inspiratory limb of the P-V loop and the volume axis (positive airflow), the area between the expiratory limb and the volume axis (negative airflow), and the area enclosed between both limbs of the loop, respectively. A graphical representation of this process is shown in Figure 3. For each patient, the average of the multiple respiratory cycles obtained in each measurement was calculated. Lastly, to obtain final inspiratory, expiratory and effective MP values (J/min), the averaged result was multiplied by the appropriate unit conversion factor and the patient’s respiratory rate.

### 2.3. Theoretical Calculation

#### 2.3.1. Inspiratory Mechanical Power

Theoretical *MP_insp_* was calculated using equations developed in the literature for volume-controlled ventilation [8,17], which estimate the area under the inspiratory limb of the P-V loop over the volume axis, as shown in Figure 3a. The formulas obtained from the literature and the alternative formulations derived in this study are summarized in Table 1. First, Gattinoni’s original formula was considered, together with the simplified version also proposed by the authors in their original work (Equations (1) and (2)) [8]. We then considered Giosa’s approximation (Equation (3)), which is derived from Gattinoni’s formulation. In Giosa’s original derivation, it is assumed that “(…) the mean value of total respiratory system resistance in mechanically ventilated patients approximates 10 cmH_2_O·s/L” [17]. While this assumption may be reasonable in adult patients, it is difficult to justify in pediatric patients, who are ventilated using endotracheal tubes of varying sizes. Therefore, in this study we also adopted what we refer to as the “generalized Giosa” equation (Equation (4)), which corresponds to the formulation obtained by stopping the derivation at the step preceding the assumption of a fixed respiratory system resistance.

#### 2.3.2. Expiratory Mechanical Power

For expiration, expiratory energy (E_exp_) was defined as the energy delivered from the lung during expiration, that is, the work performed by the respiratory system to recover its initial state. This corresponds to the area under the expiratory limb of the P-V loop with respect to the volume axis (Figure 3b). By analogy with Gattinoni’s original derivation, an equation for theoretical expiratory mechanical power was derived. This equation is based on the graphical shape of the expiratory limb of the dynamic P-V loop.

**Figure 4:**
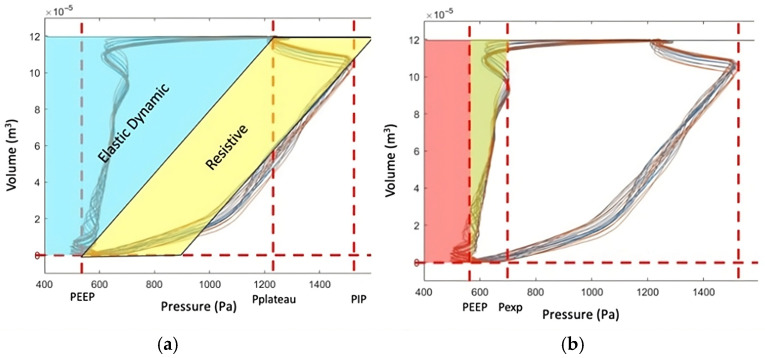
Graphical interpretation of the work performed by the lungs in inspiration and expiration: (**a**) shows Gattinoni’s approximation of the area under inspiration curve over the volume axis, considering two parts (Equation (1)), the elastic dynamic part (**blue**) and the resistive part (**yellow**); (**b**) shows the area of the expiration curve. In Equation (6), the first term is $PEEP\cdot V_{T}$, represented in this figure by the **red** rectangle, and the **green** triangle can be calculated as $1/2\cdot ∆P_{exp}\cdot V_{T}$ where ∆*P_exp_* = *P_exp_* − *PEEP*. *V_T_*: Tidal volume; *P_exp_*: expiratory pressure; *P_plateau_*: plateau pressure; *PIP*: peak inspiratory pressure.

**Figure 5:**
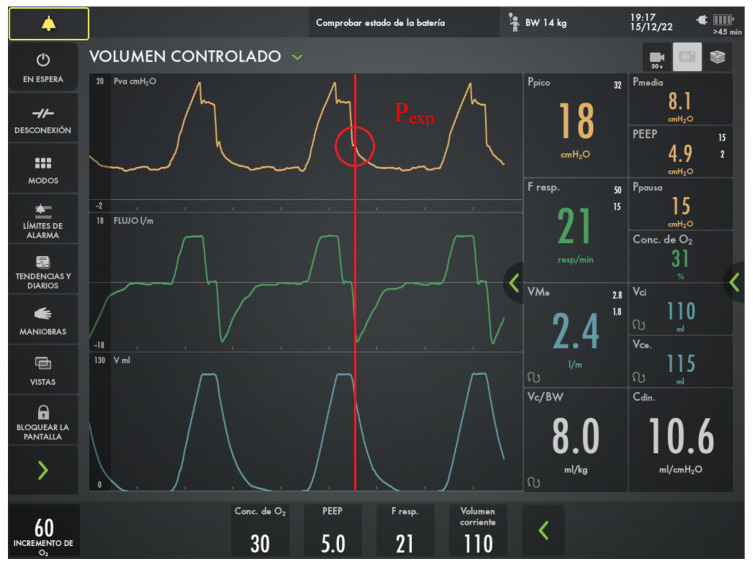
Location of *P_exp_* in the information shown by the ventilator screen. *P_exp_*: Expiratory pressure.

To compute this area, a geometrical approach was proposed by summing the area of the red rectangle and the area of the green triangle, as shown in Figure 4b. When the expiratory valve opens, expiratory flow reaches its minimum abruptly and then increases exponentially towards zero at the end of expiration. This results in an exponential decay in volume, whereas pressure along the expiratory limb of the P-V loop decreases approximately linearly throughout most of expiration. This linear behavior ends precisely when the expiratory flow reaches its minimum, as shown in the flow–time and pressure–time curves (Figure 5), and coincides with the pressure value at which an imaginary straight line, passing through the expiratory limb of the P-V loop, intersects the tidal volume value (Figure 4b). Accordingly, *P_exp_* was defined as the pressure corresponding to this point. The expression for expiratory work and its derivation is given in Equation (5).

By multiplying this expiratory energy per breath by the respiratory rate and the unit conversion factor, the expression shown in Equation (6) is obtained for the calculation of *MP_exp_* in J/min.

Furthermore, a simplified version of this equation, as shown in Equation (7), can be obtained by assuming Δ*P_exp_* = 0 when the expiratory limb of the pressure–volume loop exhibits a nearly vertical decay, as occurs in some patients.

#### 2.3.3. Effective Mechanical Power

Once *MP_insp_* and *MP_exp_* have been calculated, the final step is to compute the *MP_eff_*. According to the definition, it can be calculated as $MP_{eff}=MP_{insp}-MP_{exp}$. Consequently, several expressions for *MP_eff_* can be obtained depending on which equation of *MP_insp_* is used. In this study, we used the expression derived from Gattinoni’s simplified formula (Equation (2)) for *MP_insp_*, as it provided excellent fit with the experimental results (as detailed in Section 3). When combined with *MP_exp_* calculated from Equation (6), we obtained the resulting equation for computing *MP_eff_* (Equation (8)).

In cases where *P_exp_* cannot be estimated, *MP_eff_* can be approximated by subtracting the simplified *MP_exp_* (Equation (7)) from Gattinoni’s simplified *MP_insp_* formula (Equation (2)). This results in the simplified expression for *MP_eff_* shown in Equation (9). A geometrical comparison of both *MP_eff_* formulations is provided in Figure S1 of the Supplementary Material. Both derivations are shown below.

**Extended formula for Effective Mechanical Power (the proof to Equation (8))**

$${MP}_{eff}=0.098\;\cdot RR\;\cdot V_{T}\left(PIP-\frac{1}{2}\cdot \left(P_{Plateau}-PEEP\right)\right)-0.098\;\cdot RR\;\cdot V_{T}\left(PEEP+\frac{1}{2}\cdot \left(P_{exp}-PEEP\right)\right)=\;=0.098\;\cdot RR\;\cdot V_{T}\left[\left(PIP-\frac{1}{2}\cdot P_{Plateau}+\frac{1}{2}\cdot PEEP\right)-\left(PEEP+\frac{1}{2}\cdot P_{exp}-\frac{1}{2}\cdot PEEP\right)\right]=\;=0.098\;\cdot RR\;\cdot V_{T}\left[PIP-\frac{1}{2}\;\cdot P_{Plateau}+\frac{1}{2}\;\cdot PEEP-PEEP-\frac{1}{2}\;\cdot P_{exp}+\frac{1}{2}\;\cdot PEEP\right]=\;=0.098\;\cdot RR\;\cdot V_{T}\left[PIP-\frac{1}{2}\;\cdot P_{Plateau}-\frac{1}{2}\;\cdot P_{exp}\right]=0.098\cdot RR\cdot V_{T}\left[PIP-\frac{1}{2}\cdot \left(P_{Plateau}+P_{exp}\right)\right]$$

**Simplified formula for Effective Mechanical Power (the proof to Equation (9))**

$${MP}_{eff_{simp}}=0.098\;\cdot RR\;\cdot V_{T}\left(PIP-\frac{1}{2}\cdot \left(P_{Plateau}-PEEP\right)\right)-0.098\;\cdot RR\;\cdot V_{T}\left(PEEP\right)=\;=0.098\;\cdot RR\;\cdot V_{T}\left[PIP-\frac{1}{2}\cdot P_{Plateau}+\frac{1}{2}\cdot PEEP-PEEP\right]=0.098\;\cdot RR\;\cdot V_{T}\left[PIP-\frac{1}{2}\cdot P_{Plateau}-\frac{1}{2}\cdot PEEP\right]=\;=0.098\cdot RR\cdot V_{T}\left[PIP-\frac{1}{2}\cdot \left(P_{Plateau}+PEEP\right)\right]$$

**Figure 6:**
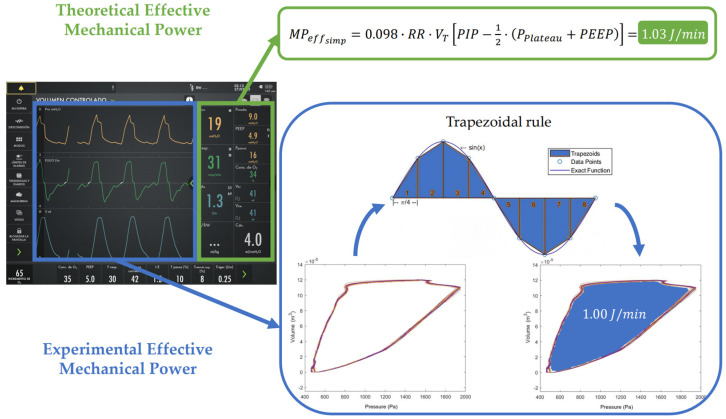
Schematic representation of the process used to obtain theoretical (**green**) and experimental (**blue**) Effective Mechanical Power values from ventilator-acquired parameters. Theoretical values are calculated by applying the corresponding equation to ventilator settings, whereas experimental values are derived from pressure–volume curves using the trapezoidal rule. ${MP}_{eff_{simp}}$: Simplified effective mechanical power; *RR*: respiratory rate (min^−1^); *V_T_*: tidal volume (L); *PIP*: peak inspiratory pressure (cmH_2_O); *P_plateau_*: plateau pressure (cmH_2_O); *PEEP*: positive end-expiratory pressure (cmH_2_O).

Figure 6 summarizes the methodology used to obtain both theoretical estimates and experimental measurements for the validation of the proposed equations.

Because the energy per unit volume seems a key determinant of VILI, MP values need to be normalized to account for differences in lung sizes among patients. Usually, lung volume measurements are normalized to ideal body weight (*IBW*), as *IBW* better reflects actual lung capacity in both adults and children [26]. A recent study further showed that normalization of MP correlates better with outcomes and disease severity in pediatric ARDS [27]. The McLaren method was used to calculate the *IBW* in children [28]. Nonetheless, to better represent the “baby lung” concept, some authors [29,30] have proposed that MP values should instead be normalized by respiratory system compliance (Crs).

In the present study, however, normalized expressions were not applied, as they are mathematically equivalent to the original equations up to a scaling factor, and would not affect the comparison of MP values across equations. Nevertheless, the normalized forms of the equations are proposed for completeness (see Table S1 in the Supplementary Material).

### 2.4. Statistical Analysis

The description of categorical variables was undertaken with percentages, and numerical variables were summarized using mean ± standard deviation or percentiles, depending on normality (Kolmogorov–Smirnov test). Population parameters were estimated with 95% confidence intervals.

Accuracy and agreement between theoretical and experimental measurements were assessed using Bland–Altman (Tukey mean-difference) plots, and correlation between methods was evaluated with scatter plots and Pearson’s correlation coefficients. All statistical analyses were conducted using MATLAB and R 4.3.1.

## 3. Results

A sample of 18 patients was studied, and their clinical characteristics are shown in Table 2. A total of 55 measurements were taken.

### 3.1. Inspiratory Mechanical Power

**Figure 7:**
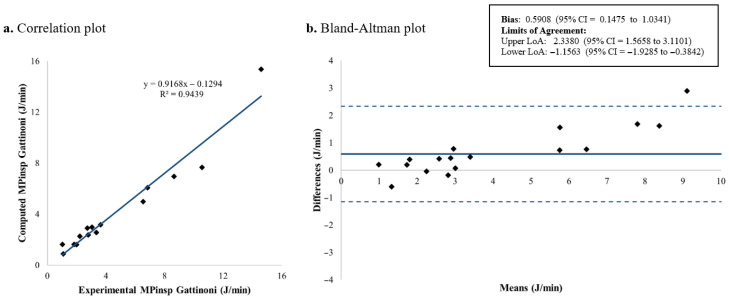
*MP_insp_* computed using Gattinoni’s original formulation: correlation and Bland–Altman plots comparing theoretical and experimental values. *MP_insp_*: Inspiratory mechanical power; LoA: limits of agreement; CI: confidence interval.

**Figure 8:**
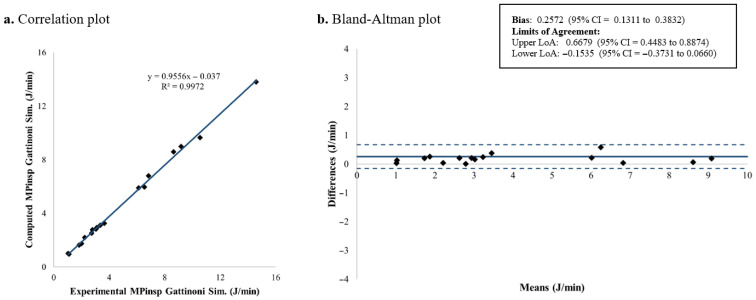
*MP_insp_* computed using Gattinoni’s simplified formulation: correlation and Bland–Altman plots comparing theoretical and experimental values. *MP_insp_*: Inspiratory mechanical power; LoA: limits of agreement; CI: confidence interval.

**Figure 9:**
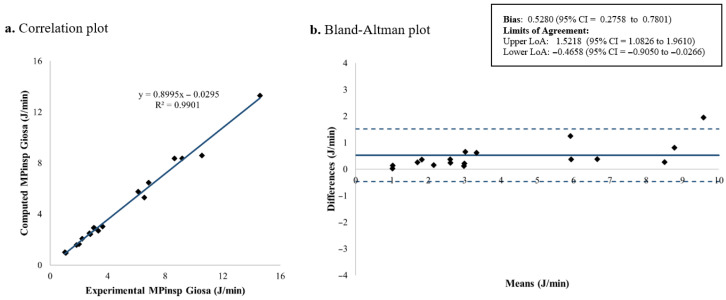
*MP_insp_* computed using Giosa’s original formulation: correlation and Bland–Altman plots comparing theoretical and experimental values. *MP_insp_*: Inspiratory mechanical power; LoA: limits of agreement; CI: confidence interval.

**Figure 10:**
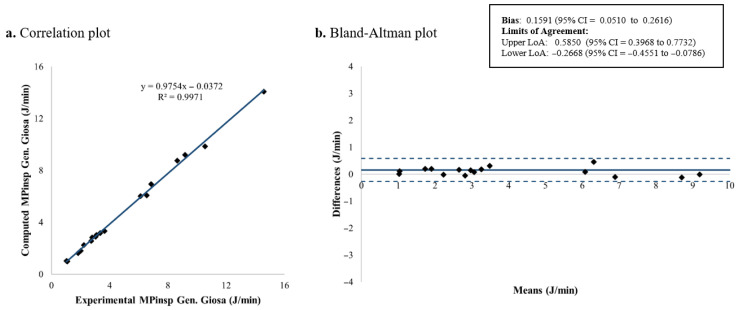
*MP_insp_* computed using Giosa’s generalized formulation: correlation and Bland–Altman plots comparing theoretical and experimental values. *MP_insp_*: Inspiratory mechanical power; LoA: limits of agreement; CI: confidence interval.

The computed and measured *MP_insp_* for each patient is shown in Table 3. Figure 7, Figure 8, Figure 9 and Figure 10 present the corresponding results. Among the four evaluated equations, the generalized Giosa equation showed the lowest mean difference for *MP_insp_*, with a mean bias of 0.1591 J/min and limits of agreement (LoA) from −0.2668 to 0.5850 J/min (Figure 10). Gattinoni’s simplified formula yielded a similar mean bias of 0.2572 J/min, with slightly narrower LoA, ranging from −0.1535 to 0.6679 J/min (Figure 8).

### 3.2. Expiratory Mechanical Power

**Figure 11:**
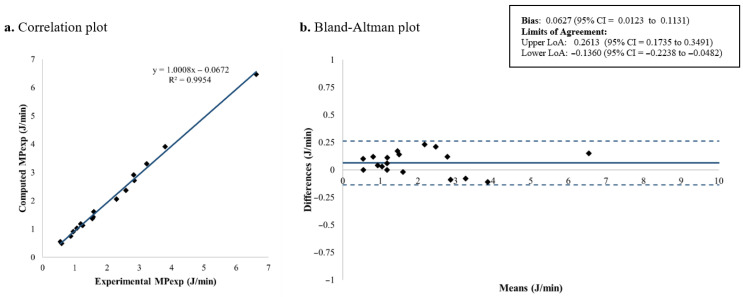
*MP_exp_* computed using the proposed extended formulation: correlation and Bland–Altman plots comparing theoretical and experimental values. *MP_exp_*: Expiratory mechanical power; LoA: limits of agreement; CI: confidence interval.

**Figure 12:**
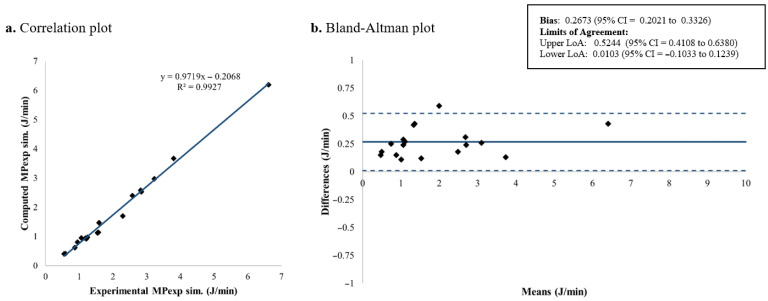
*MP_exp_* computed using the proposed simplified formulation: correlation and Bland–Altman plots comparing theoretical and experimental values. *MP_exp_*: Expiratory mechanical power; LoA: limits of agreement; CI: confidence interval.

The computed and measured *MP_exp_* for each patient is summarized in Table 4, while Figure 11 and Figure 12 illustrate the corresponding results for each proposed equation. The extended expression for *MP_exp_* demonstrated the best overall agreement, with a mean bias of 0.0627 J/min and LoA from −0.1360 to 0.2613 J/min (Figure 11). In comparison, the alternative formulation showed a mean bias of 0.2673 J/min and limits of agreement from 0.0103 to 0.5244 J/min (Figure 12). These results indicate that the extended *MP_exp_* equation provides a more accurate and consistent estimation of *MP_exp_*, while the alternative formulation tends to overestimate *MP_exp_*, likely due to the simplifications introduced in Equation (7).

### 3.3. Effective Mechanical Power

**Figure 13:**
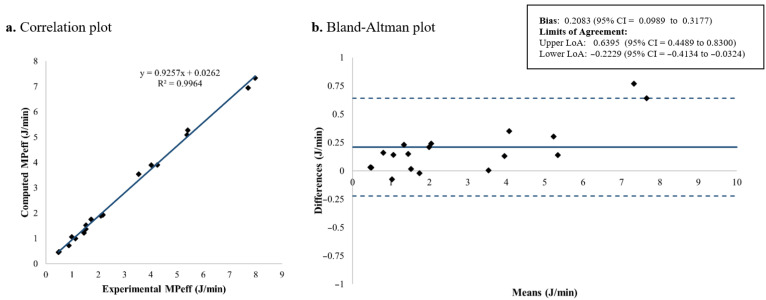
*MP_eff_* computed using the proposed extended formulation: correlation and Bland–Altman plots comparing theoretical and experimental values. *MP_eff_*: Effective mechanical power; LoA: limits of agreement; CI: confidence interval.

**Figure 14:**
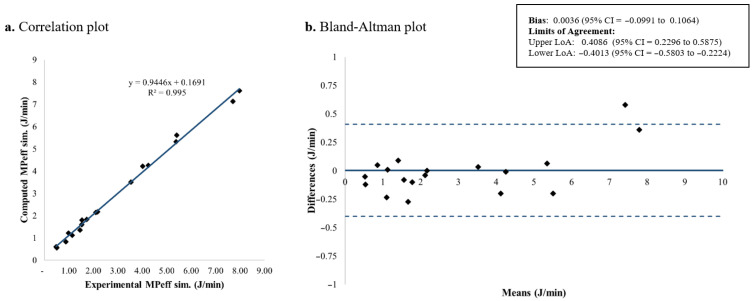
*MP_eff_* computed using the proposed simplified formulation: correlation and Bland–Altman plots comparing theoretical and experimental values. *MP_eff_*: Effective mechanical power; LoA: limits of agreement; CI: confidence interval.

Figure 13 and Figure 14 shows the Bland–Altman plots for the extended and simplified formulations of *MP_eff_*, respectively. For the extended approach, the mean difference with respect to the experimental measurements was approximately 0.2 J/min, whereas the simplified *MP_eff_* showed an estimated bias close to zero (0.0036 J/min), indicating a negligible mean difference. In both cases, the Bland–Altman limits of agreement exhibited a similar width, with an interval amplitude of around 0.8 J/min, suggesting comparable variability and acceptable agreement with the experimental results. These results show that both formulations provide reliable estimations of *MP_eff_*, with the simplified equation achieving minimal bias. The reasons underlying these differences are further addressed in Section 4.

## 4. Discussion

### 4.1. Inspiratory Mechanical Power

In this study, we validated existing formulas for inspiratory mechanical power in children under volume-controlled ventilation. Regarding *MP_insp_* calculation, four equations were evaluated: Gattinoni’s original formulation, Gattinoni’s simplified equation, Giosa’s equation and a generalized version of Giosa’s equation derived in this study. Among these equations, the generalized Giosa formulation (Equation (4), Figure 10) showed the best agreement for *MP_insp_*, followed closely by Gattinoni’s simplified equation (Equation (2), Figure 8). As expected, Giosa’s original equation (Equation (3), Figure 9) had the worst performance, since in its original description [18] it approximates Gattinoni’s formulation (Equation (1)) under the assumption of a mean airway resistance of 10 cmH_2_O·s/L in ventilated adults. In the pediatric population, airway resistance varies widely due to differences in endotracheal tube sizes (inner diameters from 2.5 to 6 mm). Previous studies have reported airway resistance values as high as 300 cmH_2_O·s/L in vitro [31,32] and 150 cmH_2_O·s/L in vivo [33,34,35,36,37,38]. In our cohort, the mean airway resistance was 24.61 ± 12.04 cmH_2_O·s/L, indicating that the main assumption underlying Giosa’s original equation does not hold in children. The slightly reduced agreement observed for Gattinoni’s original formulation (Equation (1), Figure 7) compared with its simplified version may be explained by its greater sensitivity to resistance-dependent terms, whereas the simplified equation reduces error propagation and provides improved robustness.

### 4.2. Expiratory Mechanical Power

In the second part of this study, an equation is proposed to estimate *MP_exp_*. In its derivation, the new concept of *P_exp_* was introduced. In the previous literature, D’Angelo et al. developed the concept of (P1–P2) after an inspiratory pause to represent the viscoelastic behavior of the lung stress relaxation during the inspiration [39]. Protty et al. [6] used this concept as a subrogate to measure strain rate in piglets. Reasoning by analogy, to represent the viscoelastic behavior of the lung during the expiratory phase, *P_exp_* was introduced. *P_exp_* permits the estimation of the energy involved in the expiratory limb of the P-V loop by adding a triangular area to the *PEEP* · *V_T_* square. Our analysis showed that the theoretically derived *MP_exp_* provided a highly accurate estimation of the experimental measurements, with the best agreement obtained using the non-simplified formulation (Equation (6), Figure 11). This finding is consistent with expectations, as the complete equation better preserves the actual P-V decay during expiration, allowing a more faithful representation compared with the simplified approach.

From a physics point of view, the meaning of *P_exp_* is unknown. In viscoelastic bodies (i.e., biological tissues), the deformation response induced by load (i.e., pressure) is not instantaneous. Some time is necessary for the viscous component to flow and accommodate inside the material. That phenomenon is captured in the concept of the time constant. Our conjecture is that the initial lineal shape of the pressure decay might represent pure elastic behavior, and the *P_exp_* represents the beginning of this flow accommodation of the viscous component of lung tissues. In mechanically ventilated laboratory animals, experimental evidence shows that controlling the expiratory flow to provide a constant expiratory flow attenuates VILI production [40,41], probably through the induction of a lower expiratory strain rate. Applying our concept of *P_exp_* to those animals, it is easy to understand that the pressure that corresponds to the minimum expiratory flow is very close to *PEEP*, so ∆*P_exp_* is practically zero.

### 4.3. Effective Mechanical Power

In the third part of the study, an approximation of the measurement of *MP_eff_* is proposed. In our opinion, *MP_eff_* could be the most physiologically relevant variable responsible for VILI generation. It represents the energy that cannot be recovered in the expiratory phase and must be buffered by the resilience of the tissue. If it surpasses the resilience threshold, the energy may transform into entropic losses able to induce deformities that trigger inflammation, acting as stress-raisers.

In the subsequent analysis of *MP_eff_*, the inspiratory component was calculated using Gattinoni’s simplified equation (Equation (2)). Although the generalized Giosa equation showed slightly better agreement, the difference was minimal and the parameters needed for Gattinoni’s simplified equation were more readily available, allowing consistent computation across the cohort.

**Figure 15:**
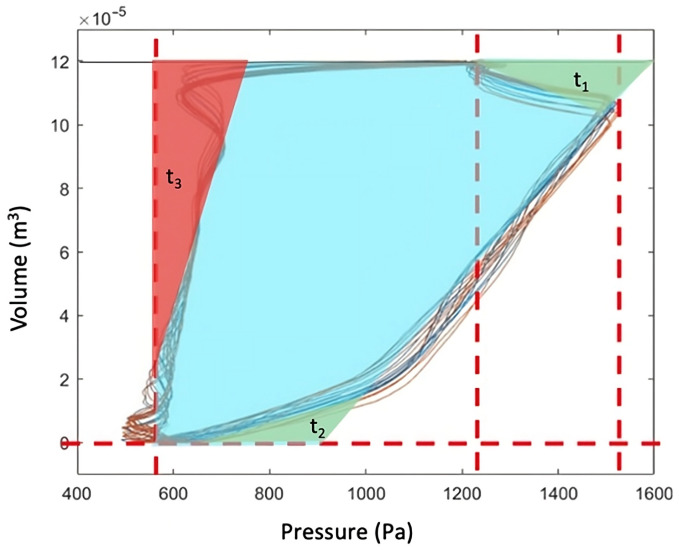
Geometrical explanation of fitting of *MP_eff_*. **Red** t_3_ triangle area compensates both **green** t_1_ + t_2_ areas.

Our analysis showed good agreement between the theoretical estimation of *MP_eff_* and its experimental measurement. As illustrated in Figure 15, and considering the geometric nature of the mathematical derivation, the effective energy per breath includes two triangular areas (t_1_ and t_2_) that contribute to an overestimation of inspiratory energy. When expiratory energy per breath is subtracted, according to the extended formula for *MP_exp_*, the red triangular area (t_3_) is additionally removed, which would be expected to reduce this overestimation and improve the estimation of *MP_eff_*.

However, contrary to this theoretical expectation, the simplified formulation of *MP_eff_* showed better agreement with experimental measurements (Equation (9), Figure 14). A possible explanation is that, in practice, *MP_insp_* tends to be underestimated (Table 3, Figure 8). Because the simplified *MP_exp_* tends to produce lower values than the full formulation, subtracting it from *MP_insp_* partially compensates for this underestimation, resulting in a more accurate overall estimation of *MP_eff_*. This compensatory effect makes the simplified *MP_eff_* a more reliable estimator of the energy actually dissipated in the lungs, despite the extended *MP_exp_* showing superior agreement when considered in isolation.

An additional advantage of the simplified *MP_eff_* formulation is its practicality: it is easier to compute, requires fewer parameters, and is less sensitive to measurement uncertainties, making it more suitable for routine clinical or experimental use.

**Figure 16:**
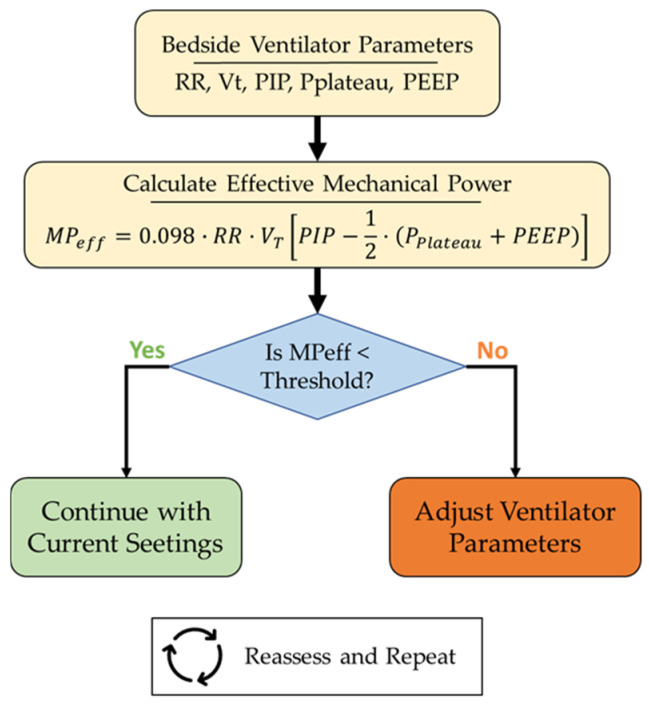
Flowchart illustrating a proposed bedside implementation of *MP_eff_* in routine PICU practice. Ventilator parameters are first obtained at the bedside; *MP_eff_* is then calculated and compared with a predefined risk threshold. If *MP_eff_* is below the threshold, current ventilatory settings are maintained; if it exceeds the threshold, ventilator parameters are adjusted to reduce lung injury risk. *MP_eff_*: Effective mechanical power; *RR*: respiratory rate (min^−1^); *V_T_*: tidal volume (L); *PIP*: peak inspiratory pressure (cmH_2_O); *P_plateau_*: plateau pressure (cmH_2_O); *PEEP*: positive end-expiratory pressure (cmH_2_O).

Figure 16 illustrates how *MP_eff_* could be implemented in routine PICU practice once a threshold associated with an increased risk of VILI has been established.

A threshold for MP associated with an increased risk of VILI must be established, especially given the wide variability in body and lung sizes in the pediatric population. Some authors propose normalizing *MP_insp_* values to make them comparable between patients. However, there is no consensus on which is the best way to normalize the MP. Two components are age-dependent (the older the child, the larger the tidal volume but the lower the respiratory rate), making it difficult to establish which variable should be used to normalize. Although *IBW* is typically used in most studies [26], a recent publication [29] based on the “baby lung” concept proposed normalization by Crs as a surrogate for functional residual capacity. They showed that normalization by *IBW* could underestimate the energy loaded per unit volume, especially in the sicker patients. Consequently, we decided to propose both normalizations in *MP_eff_* calculations (see Table S1 in the Supplementary Material). However, the results presented in the main text are based on the original, non-normalized equations, since applying a constant scaling factor does not affect the relative comparison between the formulas.

Some limitations of this study deserve attention. Due to the exploratory goal of this study, the population is heterogeneous. It is also worth mentioning that the main problem at the bedside arises from how *P_exp_* cannot be directly measured, since the information displayed on screen does not provide this value on a regular basis. In the computation of the simplified version of *MP_eff_*, *P_exp_* is not necessary, which makes it easier to use at the bedside.

More studies are needed to establish which is the best way to normalize MP and what the thresholds for *MP_insp_* or *MP_eff_* are in relation to VILI production. As we propose that *MP_eff_* could be the variable better related to VILI production, when thresholds were established, adapting ventilator parameters to the ones with lower *MP_eff_* could became a strategy to protect the lungs (Figure 16). Nevertheless, all these concepts need to be studied in different clinical scenarios before their application with real patients, especially in severe pediatric ARDS, where avoiding VILI production is the cornerstone of the therapy. It would also be important to assess the suitability of MP equations developed for the pressure-controlled mode in this age group.

## 5. Conclusions and Future Works

The equations used to calculate *MP_insp_* in adults under volume-controlled ventilation are also valid for children. Among them, the generalized Giosa equation proposed in this study showed the best agreement with experimental data, while Gattinoni’s simplified equation yielded nearly equivalent results.

Only patients ventilated in volume-controlled mode were included in this study, as this is the most commonly used ventilatory mode in our pediatric intensive care unit. For future studies, data from children ventilated in pressure-controlled mode is being collected to validate MP equations, taking into account the differences in the shape of the P-V loop.

Furthermore, we propose the concept of *MP_eff_* as the energy dissipated per minute into the lungs. Two equations (Equations (8) and (9)) were developed to estimate this energy, calculating the MP in expiration to subtract it from *MP_insp_*, yielding results that correlate very well with the experimental data. The simplified version (Equation (9)) achieves the best results.

Future investigation should focus on establishing if higher *MP_eff_*, as the energy dissipated in the lungs, correlates better than *MP_insp_* with the risk of VILI. Moreover, it is necessary to determine the threshold at which mechanical ventilation may jeopardize patients’ health, which would help clinicians to provide less damaging settings. The concept of *MP_eff_* could represent a paradigm shift, adding arguments to the theory of the viscoelastic behavior of the lung.

## Figures and Tables

**Table 1:** Different formulas obtained from the literature and their modifications. *RR*: respiratory rate (min^−1^); *V_T_*: tidal volume (L); *T_insp_*: inspiratory time (s); *PIP*: peak inspiratory pressure (cmH_2_O); *R*: resistance of the airways (cmH_2_O/L/s); *E*: elastance, the inverse of lung compliance (L/cmH_2_O); ∆*P_insp_* = *P_plateau_* − *PEEP*, where *P_plateau_* stands for the total pressure at the inspiratory pause and *PEEP* stands for the total pressure at the expiratory pause (both measured with the holding maneuvers); *F*: inspiratory Flow (L/min); E_exp_: energy per breath in expiration; *MP_exp_*: expiratory mechanical power (J/min); P-V: pressure–volume loop; *P_exp_*: pressure value that corresponds exactly with the instant in which the decay of pressure turns exponential in the P-V curve; ∆*P_exp_* = *P_exp_* − *PEEP*; *MP_eff_*: effective mechanical power; ${MP}_{eff_{simp}}$: simplified effective mechanical power.

Equation Number	Formula	Explanation
(1)	${MP}_{insp\;Gat}=0.098\cdot RR\cdot \;V_{T}\cdot \left[V_{T}\cdot \left(\frac{1}{2}\cdot E+\frac{1}{T_{insp}}\cdot R\right)+PEEP\right]$	Gattinoni’s formula
(2)	${MP}_{insp\;Gat\;Sim}=0.098\cdot RR\cdot V_{T}\cdot \left(PIP-\frac{1}{2}\cdot ∆P_{insp}\right)$	Gattinoni’s simplified formula
(3)	$MP_{insp\;Giosa}=\frac{VT\cdot RR\cdot \left(PIP+PEEP+\frac{F}{6}\right)}{20}$	Giosa’s formula
(4)	$MP_{insp\;gen\;Giosa}=\frac{VT\cdot RR\cdot (PIP+PEEP+R\cdot F)}{20\;}\;\;\;\;\;\;$	Giosa’s generalized formula. Equation (3) without the fixed-resistance assumption
(5)	$\begin{array}{ccc} E_{exp}=PEEP\cdot V_{T} & + & \frac{1}{2}\;∆P_{exp}\cdot V_{T}=V_{T}\cdot \left(PEEP+\frac{1}{2}\;∆P_{exp}\right)\\ & = & V_{T}\cdot \left(PEEP+\frac{1}{2}\;(P_{exp}-PEEP)\right)\\ & = & V_{T}\cdot \;\frac{1}{2}(PEEP+P_{exp}) \end{array}$	Energy per breath in expiration
(6)	${MP}_{exp}=0.098\;\cdot RR\;\cdot V_{T}\cdot \;\frac{1}{2}(PEEP+P_{exp})$	Expiratory mechanical power. Equation (5) multiplied by *RR* and the unit conversion factor
(7)	${MP}_{e{xp}_{simp}}=0.098\;\cdot RR\;\cdot V_{T}\cdot PEEP$	Simplified version of Equation (6) obtained assuming a vertical decay for the expiratory limb of the P-V loop (∆*P_exp_* = 0)
(8)	${MP}_{eff}=0.098\cdot RR\cdot V_{T}\left[PIP-\frac{1}{2}\cdot \left(P_{Plateau}+P_{exp}\right)\right]$	Extended Effective Mechanical Power formula. Calculation derived from Equations (2) and (6)
(9)	${MP}_{eff_{simp}}=0.098\cdot RR\cdot V_{T}\left[PIP-\frac{1}{2}\cdot \left(P_{Plateau}+PEEP\right)\right]$	Simplified Effective Mechanical Power formula. Calculation derived from Equations (2) and (7)

**Table 2:** Patients’ characteristics and ventilation parameters (median [P25: P75]). *IBW*: Ideal body weight; ENT: ear–nose–throat; PICU: Pediatric Intensive Care Unit; *V_T_*: tidal volume; *RR*: respiratory rate; *bpm*: breath per minute; *PIP*: peak inspiratory pressure; *P_plateau_*: plateau pressure; *PEEP*: positive end-expiratory pressure; ∆*P_insp_* = *P_plateau_* − *PEEP*; *P_exp_*: pressure value that corresponds exactly with the instant in which the decay of pressure turns exponential in the P-V curve; ∆*P_exp_* = *P_exp_* − *PEEP*; *FiO*_2_: inspired fraction of oxygen.

Patients		n = 18
Age (months)		21 [7.25; 81]
Sex (%male)		66.7%
Weight (kg)		10.55 [5.1; 24.9]
Heigh (cm)		87 [64.62; 125.5]
*IBW* (kg)		12.65 [7.17; 23.37]
Diagnostics (n)	Postop. Cardiac surgery	10
	Postop. Abdominal surgery	1
	Postop. ENT	1
	Septic Shock	2
	Neurocritical	4
Duration of Mechanical Ventilation (days)		4.5 [1.25; 15]
PICU length of stay (days)		12 [3.75; 22]
Hospital length of stay (days)		33 [9; 51]
Measurements		n = 55
Measurements/patient (n)		3 [3; 3.75]
*V_T_* (mL)		99.37 [47.08; 203.75]
*RR* (bpm)		21.5 [18.5; 29.5]
*PIP* (cmH_2_O)		23.17 [19; 26.75]
*P_plateau_* (cmH_2_O)		18.16 [16; 20.75]
*PEEP* (cmH_2_O)		6 [5.24; 6.86]
∆*P_insp_* [Driving Pressure] (cmH_2_O)		11.1 [10.05; 14.00]
*P_exp_* (cmH_2_O)		7.61 [7.11; 9.03]
∆*P_exp_* (cmH_2_O)		1.94 [0.99; 2.38]
*FiO*_2_ (%)		35 [30; 40]

**Table 3:** Mechanical power during inspiration *MP_insp_* (J/min) calculated with different formulas. Expressed as mean ± standard deviation.

*MP_insp_* (J/min)					
Patient	Experimental	Gattinoni (Equation (1))	Gattinoni Simplified (Equation (2))	Giosa (Equation (3))	Generalized Giosa (Equation (4))
1	3.10 ± 0.02	2.66 ± 0.01	2.94 ± 0.01	2.89 ± 0.04	3.02 ± 0.02
2	9.18 ± 0.01	7.56 ± 0.04	8.99 ± 0.02	8.37 ± 0.02	9.19 ± 0.05
3	6.12 ± 0.01	5.39 ± 0.27	5.90 ± 0.02	5.75 ± 0.02	6.03 ± 0.02
4	1.09 ± 0.02	0.89 ± 0.04	0.96 ± 0.04	0.95 ± 0.04	0.98 ± 0.04
5	2.23 ± 0.02	2.27 ± 0.00	2.19 ± 0.00	2.07 ± 0.01	2.24 ± 0.01
6	8.64 ± 0.03	6.96 ± 0.03	8.59 ± 0.02	8.37 ± 0.02	8.76 ± 0.02
7	2.00 ± 0.06	1.61 ± 0.04	1.74 ± 0.06	1.64 ± 0.07	1.80 ± 0.02
8	2.73 ± 0.01	2.91 ± 0.02	2.52 ± 0.01	2.49 ± 0.01	2.57 ± 0.01
9	14.59 ± 0.09	15.36 ± 0.34	13.8 ± 0.08	13.29 ± 0.11	14.08 ± 0.08
10	1.83 ± 0.02	1.63 ± 0.03	1.58 ± 0.02	1.57 ± 0.01	1.63 ± 0.01
11	3.35 ± 0.01	2.57 ± 0.07	3.11 ± 0.06	2.69 ± 0.04	3.17 ± 0.06
12	3.04 ± 0.00	2.97 ± 0.02	2.83 ± 0.01	2.92 ± 0.01	2.89 ± 0.01
13	6.54 ± 0.01	4.98 ± 0.06	5.96 ± 0.04	5.29 ± 0.04	6.08 ± 0.04
14	2.79 ± 0.01	2.37 ± 0.01	2.78 ± 0.01	2.42 ± 0.01	2.84 ± 0.01
15	10.55 ± 0.27	7.66 ± 0.07	9.66 ± 0.13	8.60 ± 0.02	9.86 ± 0.12
16	3.64 ± 0.02	3.16 ± 0.11	3.26 ± 0.03	3.02 ± 0.03	3.33 ± 0.04
17	6.84 ± 0.01	6.08 ± 0.04	6.80 ± 0.02	6.46 ± 0.03	6.94 ± 0.02
18	1.03 ± 0.15	1.63 ± 0.01	1.00 ± 0.01	1.00 ± 0.01	1.02 ± 0.01

**Table 4:** Mechanical power during expiration *MP_exp_* (J/min) calculated with different formulas. Expressed as mean ± standard deviation.

*MP_exp_* (J/min)			
Patient	Experimental	Extended (Equation (6))	Simplified (Equation (7))
1	1.57 ± 0.02	1.43 ± 0.01	1.14 ± 0.03
2	3.80 ± 0.01	3.91 ± 0.08	3.67 ± 0.02
3	2.58 ± 0.01	2.37 ± 0.46	2.40 ± 0.03
4	0.59 ± 0.01	0.49 ± 0.01	0.41 ± 0.01
5	1.24 ± 0.01	1.13 ± 0.01	0.97 ± 0.00
6	3.23 ± 0.01	3.31 ± 0.10	2.97 ± 0.03
7	0.87 ± 0.02	0.75 ± 0.02	0.62 ± 0.04
8	1.21 ± 0.02	1.15 ± 0.01	0.92 ± 0.00
9	6.62 ± 0.10	6.47 ± 0.10	6.19 ± 0.05
10	0.95 ± 0.01	0.91 ± 0.01	0.80 ± 0.01
11	1.18 ± 0.01	1.18 ± 0.08	0.94 ± 0.06
12	1.59 ± 0.00	1.61 ± 0.01	1.47 ± 0.02
13	2.29 ± 0.01	2.06 ± 0.05	1.70 ± 0.01
14	1.06 ± 0.00	1.03 ± 0.04	0.95 ± 0.01
15	2.84 ± 0.00	2.72 ± 0.09	2.53± 0.05
16	1.54 ± 0.02	1.37 ± 0.01	1.12 ± 0.01
17	2.82 ± 0.00	2.91 ± 0.24	2.58 ± 0.06
18	0.55 ± 0.03	0.55 ± 0.00	0.40 ± 0.01

## Data Availability

The original clinical data and ventilation curves analyzed in this study are not publicly available due to patient privacy and confidentiality. All relevant results are presented in the article. Further inquiries can be directed to the corresponding author.

## Supplementary Materials

The following supporting information can be downloaded at: https://www.mdpi.com/article/10.3390/jcm15051781/s1, Figure S1: Graphical interpretation of the effective energy per breath (E_eff_), the work carried out exclusively inside the lungs during a breath cycle, in each of both proposed formulations for the estimation of *MP_eff_*. In Equation (6) whenever *P_exp_* can be determined, the blue area shown in (a) determines the E_eff_. However, very often there are situations in which a vertical decay for expiration is observed. In this situation *P_exp_* cannot be determined and ∆*P_exp_* is near zero, so Equation (7) is applied. Then the blue area shown in (b) determines the E_effsimp_, and MPeff_simp_ is estimated. Both blue areas are very similar; Table S1. Proposed normalized equations for Effective Mechanical Power. *RR*: respiratory rate (min-1); *V_T_*: Tidal Volume (L); *PIP*: Peak Inspiratory Pressure (cmH_2_O); *P_plateau_*: Plateau pressure (cmH_2_O); *PEEP*: Positive End-Expiratory Pressure (cmH_2_O); *MP_eff_*: Effective Mechanical Power; MPeff_simp_: Simplified Effective Mechanical Power; *IBW*: Ideal Body Weight (kg); Crs: Compliance of the respiratory system (mL/cmH_2_O).

## Abbreviations

The following abbreviations are used in this manuscript:

VILI	Ventilator-Induced Lung Injury
ARDS	Acute Respiratory Distress Syndrome
VC-CMV	Volume-Controlled Continuous Mandatory Ventilation
MP	Mechanical Power
*MP_insp_*	Inspiratory Mechanical Power
E_breath_	Inspiratory Energy per breath
E_exp_	Expiratory Energy per breath
*MP_exp_*	Expiratory Mechanical Power
E_eff_	Effective Energy per breath
*MP_eff_*	Effective Mechanical Power
*P_plateau_*	Plateau pressure
*PEEP*	Positive End-Expiratory Pressure
*PIP*	Peak Inspiratory Pressure
∆*P_insp_*	*P_plateau_* − *PEEP*
*V_T_*	Tidal volume
*RR*	Respiratory Rate
*F*	Inspiratory Flow
*T_insp_*	Inspiratory Time
*R*	Airway Resistance
*P_exp_*	Expiratory Pressure
∆*P_exp_*	P_exp_ − *PEEP*
*IBW*	Ideal Body Weight
Crs	Respiratory system compliance
E	Respiratory system elastance
