# Supplementary material for: Validation of the Formulas for Mechanical Power in Children and Proposal of the Concept of “Effective Mechanical Power”
Source: J Clin Med. 2026 Feb 26;15(5):1781. doi: 10.3390/jcm15051781 (PMC12985639; doi:10.3390/jcm15051781)
Supplement: Supplementary file 1 [file jcm-15-01781-s001.zip › jcm-4129338-supplementary.pdf]

SUPPLEMENTARY MATERIAL

TITLE:

Validation of the formulas for Mechanical Power in children and proposal of the concept “Effective Mechanical Power”

AUTHOR’S NAMES

María Ferrón-Vivó[1,*], Alicia Baza-Del-Amo[2], María J. Rupérez[1], Antonio Martínez-Millana[3], Ana M. Pedrosa[1]; Roberto Tornero-Costa[3]; Yolanda Rubio-Atienza[2], Marta Aguar-Carrascosa[2], Cristina Camilo[4], Alberto Medina[5]; Vicent Modesto-i-Alapont[2]

SUPPLEMENTARY S1: Theoretical calculations

Effective Mechanical Power

Figure S1. illustrates a geometrical comparison of both expressions of MPeff. A detailed algebraic derivation of them can be found in the Methodology section in the main text.

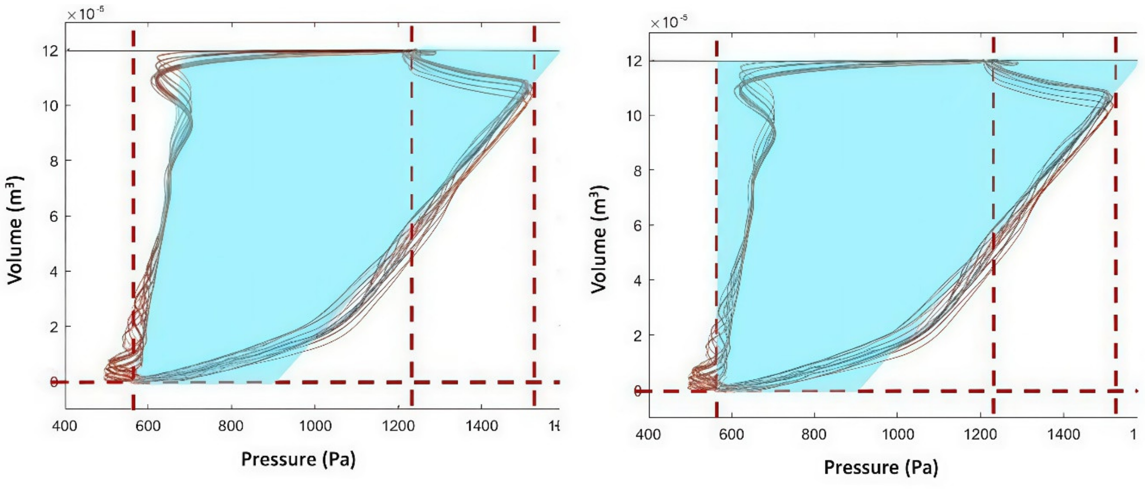

(**a**) (**b**)

Figure S1. Graphical interpretation of the effective energy per breath (Eeff), the work carried out exclusively inside the lungs during a breath cycle, in each of both proposed formulations for the estimation of MPeff. In **Eq. 6** whenever Pexp can be determined, the blue area shown in (**a**) determines the Eeff. However, very often there are situations in which a vertical decay for expiration is observed. In this situation Pexp cannot be determined and ΔPexp is near zero, so **Eq. 7** is applied. Then the blue area shown in (**b**) determines the $Eeff_{simp}$, and $MPeff_{simp}$ is estimated. Both blue areas are very similar.

In **Table S1** we present both normalization approaches for MPeff in order to facilitate the future identification of a threshold beyond which there may be an increased risk of ventilator-induced lung injury (VILI). Normalization allows comparison between patients of different ages and supports more appropriate interpatient comparisons. These normalized expressions are provided in the Supplementary Material, as normalization does not affect the validation of the equations against experimental data.

Table S1. Proposed normalized equations for Effective Mechanical Power. RR: respiratory rate (min^{-1}); V_T: Tidal Volume (L); PIP: Peak Inspiratory Pressure (cmH_2O); Pplateau: Plateau pressure (cmH_2O); PEEP: Positive End-Expiratory Pressure (cmH_2O); MPeff: Effective Mechanical Power; $MPeff_{simp}$: Simplified Effective Mechanical Power; IBW: Ideal Body Weight (kg); Crs: Compliance of the respiratory system (mL/cmH_2O).

Number	Formula	Explanation
**Eq. S1**	$nMP_{eff_{simp}} = \frac{0.098 \cdot RR \cdot V_T}{IBW}\left[PIP - \frac{1}{2} \cdot (P_{Plateau} + PEEP)\right]$	**Eq. 9** normalized by IBW
**Eq. S2**	$cMP_{eff_{simp}} = \frac{0.098 \cdot RR \cdot V_T}{Crs}\left[PIP - \frac{1}{2} \cdot (P_{Plateau} + PEEP)\right]$	**Eq. 9** normalized by Crs
